# Serum CYR61 as a potential biomarker for the diagnosis of esophagogastric junction tumor

**DOI:** 10.1042/BSR20204117

**Published:** 2021-06-18

**Authors:** Ling-Yu Chu, Jian-Yuan Zhou, Yi-Xuan Zhao, Yan-Ting Ou, Tian Yang, Yu-Hui Peng, Wang-Kai Fang, Yi-Wei Xu, Jian-Jun Xie

**Affiliations:** 1 Department of Clinical Laboratory Medicine, the Cancer Hospital of Shantou University Medical College, Shantou, China; 2 Department of Biochemistry and Molecular Biology, Shantou University Medical College, Shantou, China; 3 Department of Gastrointestinal Surgery, the First Affiliated Hospital of Shantou University Medical College, Shantou, China

**Keywords:** CYR61, diagnosis biomarker, esophagogastric junction tumor, serum biomarker

## Abstract

Background: Esophagogastric junction tumor (EGJ) is a rare but fatal disease with a rapid rising incidence worldwide in the late 20 years, and it lacks a convenient and safe method for diagnosis. The present study aimed to evaluate the potential of serum CYR61 as a biomarker for the diagnosis of EGJ tumor.

Methods: Enzyme-linked immunosorbent assay (ELISA) was used to estimate CYR61 levels in sera of 152 EGJ tumor patients and 137 normal controls. Receiver operating characteristics (ROC) was carried out to evaluate the diagnostic accuracy. The Mann–Whitney’s *U* test was used to compare the difference of serum levels of CYR61 between groups. And chi-square tests were employed to estimate the correlation of the positive rate of serum CYR61 between/among subgroups.

Results: Serum CYR61 levels were statistically lower in EGJ tumor and early-stage EGJ tumor patients than those in normal controls (*P*<0.0001). The sensitivity, specificity and the area under the curve (AUC) of this biomarker in EGJ tumor were 88.2%, 43.8% and 0.691, respectively, and those for early stage of EGJ tumor were 80.0%, 66.4% and 0.722, respectively. Analyses showed that there was no correlation between the clinical data and the levels of CYR61 (*P*>0.05).

Conclusion**:** The present study showed that CYR61 might be a potential biomarker to assist the diagnosis of EGJ tumor.

## Introduction

Esophagogastric junction (EGJ) tumor is a rare but fatal disease with a rapid rising incidence worldwide in the late 20 years [1]. Studies have shown that incidence rate of EGJ tumor in China is higher than that in western countries [2–4]. Adenocarcinoma is the most common histology type, accounting for more than 90% of all EGJ tumors [5,6]. Due to the lack of epidemiological available data and public supervision, the diagnosis of EGJ tumor has always been complex. So far, the primary strategy in clinic of early detection for EGJ tumor is endoscopy that is invasive, unacceptable to some patients and proved to have side effects [7]. In western countries, patients with EGJ tumors are always diagnosed as advanced cancer with poor prognosis because of the nonspecific symptoms at early stage [1]. What’s more, despite a variety of treatment options, such as radical surgery, chemotherapy and radiotherapy, patients with EGJ tumor still appear extremely low survival rate [8–10]. Five-year overall survival (OS) rates with surgery alone are gloomy at approximately 25% [11]. Thus, a reliable and sensitive early detection method that has clinical value for effective treatment and improving the prognosis of patients is urgently needed for EGJ tumor patients.

CCN1/CYR61 is a protein from CCN family, which contains five parts: an N-terminal secretory signal peptide and four functional domains: an insulin-like growth factor-binding protein domain (IGFBP), a Von Willebrand factor domain (VWC), a thrombospondin type-1 repeat module (TSP-1) and a CT [12]. It can be induced rapidly by growth factors. As an angiogenic inducer that can promote tumor growth and vascularization, it plays an important part in promoting cell survival, proliferation, differentiation, angiogenesis and inducing apoptosis and senescence by binding directly to the integrins and heparin sulfate proteoglycans or activating multiple signaling transduction pathways [13–15]. This suggests that CYR61 might be useful as a biomarker or therapeutic target in certain diseases. Some studies have indicated that high expression of CYR61 was related to colorectal cancer [16], prostate cancer [17,18], ovarian cancer [19], glioma [20], osteosarcoma [21], gastric cancer [22] and breast cancer [23,24]. Meanwhile, it was proved that the expression of CYR61 reduced in high-grade chondrosarcomas [25], advanced gastric cancers [25], endometrial cancer [26] and lung cancer [27]. What’s more, multiple studies showed that CYR61 could be a metastatic biomarker for prediction of poor prognosis of EGJ tumors [28] and a potential diagnosis biomarker for colorectal cancer [29] and endometrial cancer [15]. However, the application of serum CYR61 as a clinical biomarker in the diagnosis of EGJ tumor patients has rarely been reported. The aim of our study is to examine the use of serum CYR61 as a potential biomarker for the diagnosis of EGJ tumor.

## Materials and methods

### Study sample

In the present study, we set the sample size required for the EGJ tumor group and the normal control group to be equal. In order to estimate the sample size, we calculate it according to the following formula [30,31].

$$\text{Sample}\;\text{size}(\text{n})\text{based}\;\text{on}\;\text{Sensitivity}=\frac{\left[{\text{Z}}_{1-\alpha /3}^{2}\times {\text{S}}_{\text{N}}\times (1-{\text{S}}_{\text{N}})\right]}{\left({\text{L}}^{2}\times \text{P}\right)}$$

$$\text{Sample}\;\text{size}(\text{n})\text{based}\;\text{on}\;\text{Specificity}=\frac{\left[{\text{Z}}_{1-\alpha /2}^{2}\times {\text{S}}_{\text{P}}\times (1-{\text{S}}_{\text{P}})\right]}{\left[{\text{L}}^{2}\times (1-\text{P})\right]}$$

*Z*_1-α/2_ is the value of *Z* when the cumulative probability in the normal distribution is equal to α/2, When α is 0.05, *Z*_1-α/2_ is 1.96, and when α is 0.01, *Z*_1-α/2_ is 2.58. *L* is the width of the 95% interval of sensitivity or specificity that we allow, which is artificially designated by the researcher, and is generally set at 0.03–0.1. Here, we set the allowable error (*L*) as 0.1 and α as 0.05. In the preliminary experiment, we concluded that the sensitivity (SN) of cyr61 for EGJ diagnosis is 0.4, the specificity (SP) is 0.9, and the disease prevalence (*P*) is 0.6.

When we use the sensitivity to estimate the sample size, according to the formula:

$$\text{N}1=\frac{\left[1.96^{2}\times 0.4\times (1-0.4)\right]}{\left(0.1^{2}\times 0.6\right)}\approx 154$$

When we use the specificity to estimate the sample size, according to the formula:

$$\text{N}2=\frac{\left[1.96^{2}\times 0.9\times (1-0.9)\right]}{\left[0.1^{2}\times (1-0.6)\right]}\approx 86$$

Because N1 > N2, according to the principle of which general election, and taking N1 as a reference, it reminds us that we need to include at least 154 research subjects. For some other reasons, the actual sample size we collected was 152 cases in the EGJ tumor group and 137 cases in the normal control group. Among the 152 serum samples from EGJ tumor patients, 81 were diagnosed at the First Affiliated Hospital of Shantou University Medical College and 71 were diagnosed at Cancer Hospital of Shantou University Medical College from October 2017 to December 2019. And the 137 serum samples from normal controls were selected from the First Affiliated Hospital of Shantou University Medical College. All the normal samples were healthy subjects without cancer signs. After being coagulated at room temperature for 30 min and centrifuged at 1250***g*** for 5 min, all the serum samples were stored at −80°C until use.

The diagnosis of EGJ tumor was confirmed by histopathology and the tumor stage was referred to the eight edition of the American Joint Committee on Cancer (AJCC) Cancer Staging Manual [32]. In the present study, EGJ tumor with TNM stage 0 + I + IIA was defined as early-stage EGJ tumor.

### Enzyme-Linked Immunosorbent Assay (ELISA) for CYR61

The Serum concentrations of CYR61 were tested by ELISA Kit based on manufacturer’s directions. Reagents, samples and standards were prepared as instructed. The CYR61 standard concentrations for standard curve were 0, 78, 156, 312, 625, 1250, 2500 and 5000 pg/ml, respectively. It was proved in our preliminary experiments that the most appropriate dilution ratio was 1:1. After adding 100 μl standards and serum samples (a 2-fold dilution) per well, the 96-well plate were incubated for 2 h at 37°C. Then the liquid was removed and 100 μl of biotin-antibody (1×) was added to each well and incubated for 1 h at 37°C followed by washing the plate for three times using microplate washer with water buffer. Before accomplishing the same washing procedure for another five times, 100 μl of HRP-avidin was added to each well and incubated for 1 h at 37°C. After adding 90 μl TMB substrate to each well, the plate was incubated for 20 min at 37°C protected from light. Color formation was stopped by 50 μl Stop Solution, and the optical density (OD) value was read at wavelength of 450 and 590 nm on a plate microplate reader within 5 min. Corresponding concentrations were converted from OD values using standard curve method (Table 1). Each serum sample was tested twice and the average value was taken for analysis.

**Table 1:** Participant information and clinicopathological characteristics

Group	EGJ tumor patients (*n*=152)	Normal controls (*n*=137)
**Age, years**		
**Mean** ± **SD**	64.34 ± 9.578	48.32 ± 12.529
**Range**	22-93	24-81
**Gender**		
**Male**	125	65
**Female**	27	72
**Smoke**		
**Yes**	25	
**No**	72	
**Unknown**	55	
**TNM Stage**		
**0**	2	
**I**	13	
**II**	6	
**III**	29	
**IV**	44	
**Unknown**	58	
**Histological stage**		
**High (Grade 1)**	5	
**Middle (Grade 2)**	19	
**Low (Grade 3)**	27	
**Unknown**	101	
**Depth of tumor invasion (T staging)**		
**Tis**	2	
**T1**	12	
**T2**	9	
**T3**	22	
**T4**	45	
**Unknown**	62	
**Regional lymph nodes (N staging)**		
**N0**	27	
**N1**	19	
**N2**	16	
**N3**	27	
**Unknown**	63	
**Metasstasis**		
**M0**	83	
**M1**	9	
**Unknown**	60	

Abbreviation: EGJ tumor, esophagogastric junction tumor.

### Statistical analysis

With Microsoft Excel, SPSS (version 20.0), GraphPad Prism 8.0 and Sigma Plot 10.0 software, data analyses were performed statistically. The Mann–Whitney’s *U* test was used to compare the difference of serum levels of CYR61 between EGJ tumor group and normal group, early-stage EGJ tumor group and normal group. And chi-square tests were used to estimate the correlation between different clinical data and the positive rate, and the correlation between different groups. Plotting ROC curves and calculating the area under ROC curves (AUC) [33] with 95% confidence interval were used to analyze the accuracy of diagnostic value. The optimum cut-off values were obtained from the Youden’s indexes of the ROC curves and the maximum indexes were calculated by the sum of sensitivity and specificity minus 1. And sensitivity, specificity, positive predictive values (PPV), negative predictive values (NPV), false positive rate (FPR), false negative rate (FNR), positive likelihood ratio (PLR) and negative likelihood ratio (NLR) were calculated using the optimum cut-off values to further evaluate the diagnostic value. *P*<0.05 (two-sided) was considered as statistically significant in all the analyses.

## Results

### The levels of serum CYR61 in EGJ tumor patients and normal controls

**Figure 1:**
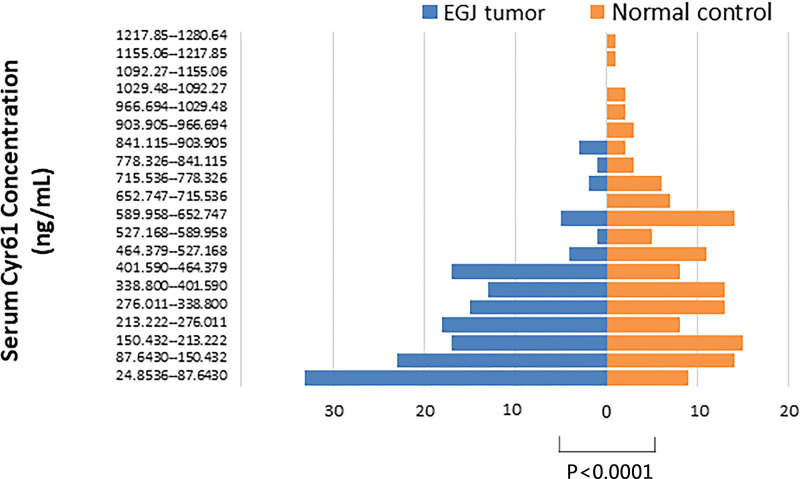
Bar chart of concentration of CYR61 from EGJ tumor serum and normal serum The diagram of EGJ tumor (*n*=152) is in blue; the one of normal control (*n*=137) is in orange. The lowest concentration was 24.85 ng/ml in EGJ tumor and the highest one was 1280.64 ng/ml in normal control. The concentration was divided into 20 sections equally. EGJ tumor stands for more histogram volume on lower concentration while normal control accounts for more on higher concentration.

**Figure 2:**
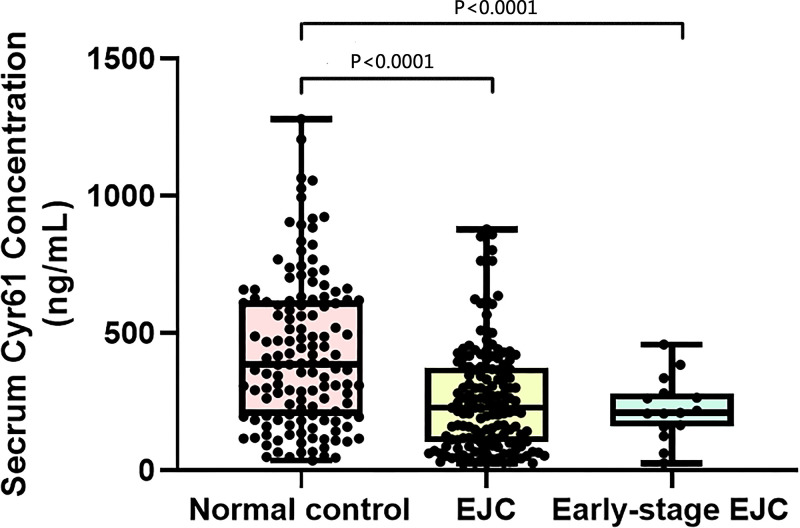
Scatter plots and box plots of concentration of CYR61 from EGJ tumor serum, early-stage EGJ tumor serum and normal serum Every sample of the concentration of serum CYR61 in three groups were shown in scatter plots and box plots (*P*<0.0001). The central line is median. It showed the degree of dispersion. The lines up and down are the extremum; EGJ tumor, esophagogastric junction tumor. CYR61 is a protein from CCNs family.

In our study, 289 serum samples were tested, including EGJ tumor group (*n*=152) and normal control group (*n*=137), with the mean age of 64 years old and 48 years old respectively. (Table 1) The mean concentration of serum CYR61 in EGJ tumor group (*n*=152), early-stage EGJ tumor group (*n*=15) and normal group (*n*=137) was 258.515 ± 191.736 ng/ml, 225.146 ± 114.316 ng/ml, and 429.115 ± 273.432 ng/ml, respectively (Table 2). There was a difference between the distribution of EGJ tumor group and normal control group. The EGJ tumor group accounted for more histogram volume at low concentration while normal group accounted for more at high concentration (Figure 1). More intuitive distribution and dispersion could be seen in combined scatter plot and box plot (Figure 2). Confirmed by statistics, the level of serum CYR61 in EGJ tumor group was lower than that in normal controls (*P*<0.0001), and there was also a significant difference between early-stage EGJ tumor group and normal control group (*P*<0.0001). (Table 2)

**Table 2:** Comparison between three groups

	*N*	Mean ± SD	*P* value	95%CI
**EGJ tumor**	152	258.515 ± 191.736	*(<0.0001)	227.788–289.243
**Early-stage EGJ tumor (0+I+IIA)**	15	225.146 ± 114.316	*(<0.0001)	161.840–288.453
**Normal controls**	137	429.115±273.432		382.917–475.312

*compared with normal controls; EGJ tumor, esophagogastric junction tumor.

### The diagnostic value of serum CYR61 in EGJ tumor and early-stage EGJ tumor

**Figure 3:**
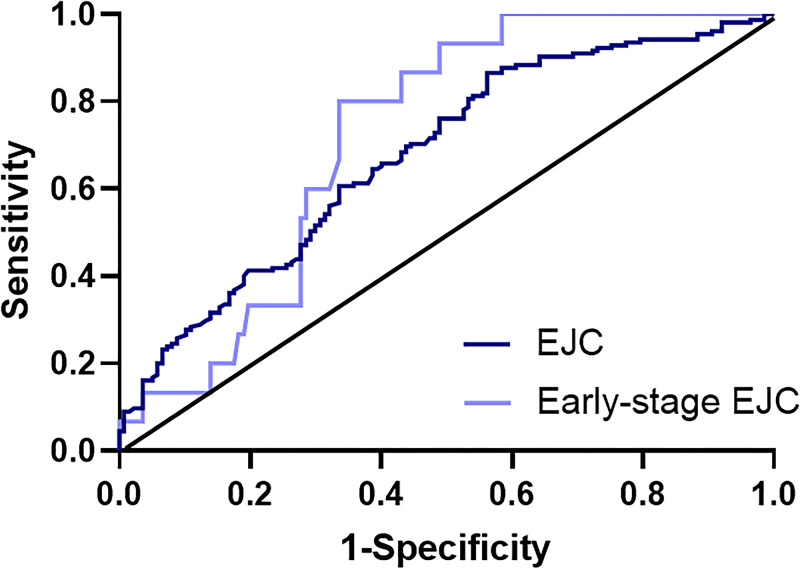
ROC curve analysis in the diagnosis of EGJ tumor and early-stage EGJ tumor Two groups versus normal controls group are in different colors. The area under the red line is 0.5 for reference; ROC curve, receiver operating characteristic curve; EGJ tumor, esophagogastric junction tumor.

ROC curve was established to evaluate the diagnostic value of CYR61 in EGJ tumor. According to the ROC curve of EGJ tumor group and normal group (Figure 3), AUC was 0.691, and the optimized cutoff value of 445.708 ng/ml was selected with specificity of 43.8% and sensitivity of 88.2%. For early-stage EGJ tumor group with AUC of 0.722 and the cutoff value of 281.947 ng/ml, specificity and the sensitivity were 66.4% and 80.0%, respectively. And the positive rates of EGJ tumor and early-stage EGJ tumor were much higher than that of the normal controls (Table 2). In order to better explain the clinical value, more relevant indicators were calculated and the results were displayed with 95% confidence interval, including false positive rate, false negative rate, positive predictive value, negative predictive value, positive likelihood ratio and negative likelihood ratio (Table 3).

**Table 3:** Evaluation of the detection value of CYR61 in the diagnosis of EGJ tumor

	AUC	SEN	SPE	FPR	FNR	PPV	NPV	PLR	NLR
**EGJ tumor vs. NC**	0.691	88.2% (81.7–92.6%)	43.8% (35.4–52.5%)	56.2% (47.5–64.6%)	11.8% (7.4–18.3%)	63.5% (56.6–69.9)	76.9% (65.8–85.5%)	1.57 (1.34–1.84)	0.27 (0.17–0.42)
**Early-stage EGJ tumor vs. NC**	0.722	80.0% (51.4–94.7%)	66.4% (57.8–74.1%)	33.6% (25.9–42.2%)	20.0% (5.3–48.6%)	20.7% (11.6–33.7%)	96.8% (90.3–99.2%)	2.38 (1.68–3.36)	0.30 (0.11–0.83)

95% CI were given in brackets for each group. AUC, area under the ROC curve; EGJ tumor, esophagogastric junction tumor; FNR, false negative rate; FPR, false positive rate; NC, normal controls; NLR, negative likelihood ratio; NPV, negative predictive value; PLR, positive likelihood ratio; PPV, positive predictive value; SEN, sensitivity; SPE, specificity.

### Correlation between serum concentration of CYR61 and clinical data in patients with EGJ tumor

The association between serum CYR61 level of patients with EGJ tumor and clinical data variables was shown in Table 4. There was no statistically significant correlation between positive rate of serum CYR61 and clinical data, including age, gender, smoking status, depth of tumor invasion, lymph node status, metastasis, histological grade and early-stage or advanced-stage of EGJ tumor (all *P*>0.05).

**Table 4:** Correlation between CYR61 and clinical data in EGJ patients

	*N*	Positive	%	95%CI	*P*
**Age**					0.823
**≥60**	115	101	87.8%	80.1–92.9	
**<60**	37	33	89.2%	73.6–96.4	
**Gender**					0.598
**Male**	125	111	88.8%	81.6–93.5	
**Female**	27	23	85.2%	65.4–95.1	
**Smoke**					0.780
**Yes**	25	21	84.0%	63.1–94.7	
**No**	72	64	88.9%	78.7–94.7	
**Unknown**	55	49	89.1%	77.1–95.5	
**T**					0.819
**Tis+T1+T2**	23	21	91.3%	70.5–98.5	
**T3+T4**	67	58	86.6%	75.5–93.3	
**Unknown**	62	55	88.7%	77.5–95.0	
**N**					0.360
**N0**	27	22	81.5%	61.3–93.0	
**N1+N2+N3**	62	57	91.9%	81.5–97.0	
**Unknown**	63	55	87.3%	76.0–94.0	
**M**					0.119
**M0**	83	74	89.2%	79.9–94.6	
**M1**	9	6	66.7%	30.9–91.0	
**Unknown**	60	54	90.0%	78.8–95.9	
**Grade**					0.818
**G1**	5	5	100.0%	46.3–100	
**G2**	19	17	89.5%	65.5–98.2	
**G3**	27	23	85.2%	65.4–95.1	
**Unknown**	101	89	88.1%	79.8–93.4	
**TNM stage**					0.658
**Early**	15	14	93.3%	66.0–99.7	
**Advanced**	79	68	86.1%	76.0–92.5	
**Unknown**	58	52	89.7%	78.2–95.7	

Abbreviations: EGJ tumor, esophagogastric junction tumor.

## Discussion

At present, the diagnostic examination choice for EGJ tumor is upper esophagogastroduodenoscopy [1,5], an invasive method with serious side effects, which is not suitable for the screening and detection of asymptomatic population. With the development of clinical medicine, the detection of serum tumor biomarkers, as a painless, convenient, and most importantly, non-invasive detection method, has been widely developed in clinical diagnosis. Tumor biomarkers are a kind of substances reflecting the existence of tumors. When these substances reach a certain level *in vivo*, they can predict the existence of some tumors, which makes it possible to diagnose EGJ tumor early [34]. In this regard, our study found that CYR61 might be a potential biomarker for the diagnosis of EGJ tumor.

CYR61 plays an important role in tumor angiogenesis, tumor cell proliferation, apoptosis and tumor metastasis, which closely participates in the occurrence and development of tumors [13–15]. An increasing number of studies have proved CYR61 to be a metastatic biomarker for prediction of prognosis in osteosarcoma [21], gastric cancer [22], colorectal cancer [16], laryngeal tumor [35], ovarian carcinoma [36] and prostate cancer [37]. As for EGJ tumors, a study has suggested that CYR61 might serve as a metastatic predictor of poor prognosis and provide a potential molecular target for anti-metastatic therapy of EGJ tumor [28]. In addition, many other studies have shown that CYR61 could also act as a diagnosis predictor in patients with colorectal cancer [29] and endometrial cancer [15]. However, CYR61 as a potential biomarker for diagnosis of EGJ tumor has not yet been reported. In the present study, ROC results showed that AUC was 0.691, specificity was 43.8%, and sensitivity was 88.2%, suggesting the diagnostic value of serum CYR61 for EGJ tumor. Similar results could also be demonstrated in early EGJ tumor. Taking other diagnostic evaluation indices into consideration contributes to better understanding of the diagnostic value of serum CYR16 in EGJ tumor, including false positive rate (FPR) of 56.2% (95%CI: 47.5–64.6%), false negative rate (FNR) of 11.8% (95%CI: 7.4–18.3%), positive predictive value (PPV) of 63.5% (95%CI: 56.6–69.9%), negative predictive value (NPV) of 76.9% (95%CI: 65.8–85.5%), positive likelihood ratio (PLR) of 1.57 (95%CI: 1.34–1.84) and negative likelihood ratio (NLR) of 0.27 (95%CI: 0.17–0.42). Meanwhile, in the present study, the serum CYR61 concentration in EGJ tumor was shown significantly lower than that in healthy control group (*P*<0.001), which was inconsistent with the results of high expression in the study of colon cancer, esophageal cancer and many other cancers. Therefore, we infer that the expression pattens of CYR61 differ in different types of tumors, as well as different histopathological types may lead to the difference, thereby CYR61 has certain significance for the differential diagnosis of tumors and a broad application prospect as a diagnostic biomarker of tumors.

However, there are still some limitations in the present study. It remains open to be discussed and improved. Relatively low specificity may limit the clinical application of CYR61 in the screening of asymptomatic early EGJ tumor patients, so a single detection of serum CYR61 is unable to meet the clinical demands. As reported, compared with single biomarker, combined detection of multiple serum proteins could help improve the sensitivity or specificity of gastrointestinal cancer screening [38], which provides us with a new research direction: CYR61 could be combined with other tumor markers or even other tests to diagnose EGJ tumor. Because the age and sex of normal control group were mismatching with that of EGJ tumor cases, further study could be carried out according to the corresponding age and sex. However, the *P* value of the variance test between the age and the concentration of serum CYR61 was 0.153 and the one between the sex and the concentration of serum CRY61 was 0.249, which showed that CYR61 has no significant relationship with the age and the sex. So, the age and the sex bias between the two groups could be reduced. In addition, due to the low clinical incidence of EGJ tumor and difficulty of diagnosing EGJ tumor as early cancer, the sample size of our study is small. Besides, incomplete clinical data and single center study are also likely bias. Our conclusion only suggested the possibility of CYR61 being a potential biomarker in the early detection of EGJ tumor. We hope further in-depth studies with large sample size, complete clinical information and well-matched age and sex controls in multiple institutions could be conducted, which could help better evaluate the diagnostic value of CYR61 as a biomarker.

## Conclusion

In summary, our study evaluated the relationship between serum CYR61 and EGJ tumor, and proved that serum CYR61 could be a potential biomarker in the early detection of EGJ tumor.

## Data Availability

The data were collected and saved in hospital’s medical history management center. Due to the legitimate protection of patients’ privacy, our information is not available on public or any private websites, but is available from the corresponding author on reasonable request.

## Abbreviations

AJCC: American Joint Committee on Cancer
AUC: area under the ROC curve
CI: confidence interval
CYR61: cysteine-rich angiogenic inducer 61
EGJ tumor: esophagogastric junction tumor
ELISA: enzyme-linked immunosorbent assay
FNR: false negative rate
FPR: false positive rate
NLR: negative likelihood ratio
NPV: negative predictive value
OD: optical density
PLR: positive likelihood ratio
PPV: positive predictive value
ROC curve: receiver operating characteristic curve

## References

[1] Chevallay M., Bollschweiler E., Chandramohan S.M. et al. (2018) Cancer of the gastroesophageal junction: a diagnosis, classification, and management review. Ann. N. Y. Acad. Sci. 1434, 132–138 10.1111/nyas.13954 30138540

[2] Bray F., Ferlay J., Soerjomataram I. et al. (2018) Global cancer statistics 2018: GLOBOCAN estimates of incidence and mortality worldwide for 36 cancers in 185 countries. CA Cancer J. Clin. 68, 394–424 10.3322/caac.21492 30207593

[3] Liu K., Yang K., Zhang W. et al. (2016) Changes of esophagogastric junctional adenocarcinoma and gastroesophageal reflux disease among surgical patients during 1988-2012: a single-institution, high-volume experience in China. Ann. Surg. 263, 88–95 10.1097/SLA.0000000000001148 25647058 PMC4679348

[4] Urabe M., Ushiku T., Shinozaki-Ushiku A. et al. (2018) Adenocarcinoma of the esophagogastric junction and its background mucosal pathology: a comparative analysis according to Siewert classification in a Japanese cohort. Cancer Med. 7, 5145–5154 10.1002/cam4.1763 30239168 PMC6198208

[5] Okereke I.C. (2017) Management of gastroesophageal junction tumors. Surg. Clin. North Am. 97, 265–275 10.1016/j.suc.2016.11.004 28325186

[6] Azari F.S. and Roses R.E. (2019) Management of early stage gastric and gastroesophageal junction malignancies. Surg. Clin. North Am. 99, 439–456 10.1016/j.suc.2019.02.008 31047034

[7] Xu Y.W., Peng Y.H., Xu L.Y. et al. (2019) Autoantibodies: Potential clinical applications in early detection of esophageal squamous cell carcinoma and esophagogastric junction adenocarcinoma. World J. Gastroenterol. 25, 5049–5068 10.3748/wjg.v25.i34.5049 31558856 PMC6747294

[8] Li J., Qin S., Xu J. et al. (2016) Randomized, double-blind, placebo-controlled phase III trial of apatinib in patients with chemotherapy-refractory advanced or metastatic adenocarcinoma of the stomach or gastroesophageal junction. J. Clin. Oncol. 34, 1448–1454 10.1200/JCO.2015.63.5995 26884585

[9] Anderson L.A., Tavilla A., Brenner H. et al. (2015) Survival for oesophageal, stomach and small intestine cancers in Europe 1999-2007: Results from EUROCARE-5. Eur. J. Cancer 51, 2144–2157 10.1016/j.ejca.2015.07.026 26421818 PMC5729902

[10] Siegel R.L., Miller K.D. and Jemal A. (2017) Cancer Statistics, 2017. CA Cancer J. Clin. 67, 7–30 10.3322/caac.21387 28055103

[11] Ychou M., Boige V., Pignon J.P. et al. (2011) Perioperative chemotherapy compared with surgery alone for resectable gastroesophageal adenocarcinoma: an FNCLCC and FFCD multicenter phase III trial. J. Clin. Oncol. 29, 1715–1721 10.1200/JCO.2010.33.0597 21444866

[12] Lau L.F. and Lam S.C. (1999) The CCN family of angiogenic regulators: the integrin connection. Exp. Cell. Res. 248, 44–57 10.1006/excr.1999.4456 10094812

[13] Lau L.F. (2011) CCN1/CYR61: the very model of a modern matricellular protein. Cell. Mol. Life Sci. 68, 3149–3163 10.1007/s00018-011-0778-3 21805345 PMC3651699

[14] Babic A.M., Kireeva M.L., Kolesnikova T.V. et al. (1998) CYR61, a product of a growth factor-inducible immediate early gene, promotes angiogenesis and tumor growth. Proc. Natl. Acad. Sci. U. S. A. 95, 6355–6360 10.1073/pnas.95.11.6355 9600969 PMC27701

[15] Yang R., Chen Y. and Chen D. (2018) Biological functions and role of CCN1/Cyr61 in embryogenesis and tumorigenesis in the female reproductive system (Review). Mol. Med. Rep. 17, 3–10 29115499 10.3892/mmr.2017.7880 PMC5780141

[16] Jeong D., Heo S., Ahn T.S. et al. (2014) Cyr61 expression is associated with prognosis in patients with colorectal cancer. BMC Cancer 14, 164 10.1186/1471-2407-14-164 24606730 PMC3975645

[17] Terada N., Kulkarni P. and Getzenberg R.H. (2012) Cyr61 is a potential prognostic marker for prostate cancer. Asian J. Androl. 14, 405–408 10.1038/aja.2011.149 22343491 PMC3472413

[18] D'Antonio K.B., Toubaji A., Albadine R. et al. (2010) Extracellular matrix associated protein CYR61 is linked to prostate cancer development. J. Urol. 183, 1604–1610 10.1016/j.juro.2009.12.006 20172544 PMC3349619

[19] Gery S., Xie D., Yin D. et al. (2005) Ovarian carcinomas: CCN genes are aberrantly expressed and CCN1 promotes proliferation of these cells. Clin. Cancer Res. 11, 7243–7254 10.1158/1078-0432.CCR-05-0231 16243794

[20] Xie D., Yin D., Tong X. et al. (2004) Cyr61 is overexpressed in gliomas and involved in integrin-linked kinase-mediated Akt and beta-catenin-TCF/Lef signaling pathways. Cancer Res. 64, 1987–1996 10.1158/0008-5472.CAN-03-0666 15026334

[21] Liu Y., Zhang F., Zhang Z. et al. (2017) High expression levels of Cyr61 and VEGF are associated with poor prognosis in osteosarcoma. Pathol. Res. Pract. 213, 895–899 10.1016/j.prp.2017.06.004 28647210

[22] Zhao Z.S., Li L., Wang H.J. et al. (2011) Expression and prognostic significance of CEACAM6, ITGB1, and CYR61 in peripheral blood of patients with gastric cancer. J. Surg. Oncol. 104, 525–529 10.1002/jso.21984 21618249

[23] Tsai M.S., Bogart D.F., Li P. et al. (2002) Expression and regulation of Cyr61 in human breast cancer cell lines. Oncogene 21, 964–973 10.1038/sj.onc.1205131 11840342

[24] Hellinger J.W., Huchel S., Goetz L. et al. (2019) Inhibition of CYR61-S100A4 axis limits breast cancer invasion. Front. Oncol. 9, 1074 10.3389/fonc.2019.01074 31709177 PMC6819319

[25] Maeta N., Osaki M., Shomori K. et al. (2007) CYR61 downregulation correlates with tumor progression by promoting MMP-7 expression in human gastric carcinoma. Oncology 73, 118–126 10.1159/000121000 18337624

[26] Chien W., Kumagai T., Miller C.W. et al. (2004) Cyr61 suppresses growth of human endometrial cancer cells. J. Biol. Chem. 279, 53087–53096 10.1074/jbc.M410254200 15471875

[27] Chen P.P., Li W.J., Wang Y. et al. (2007) Expression of Cyr61, CTGF, and WISP-1 correlates with clinical features of lung cancer. PLoS One 2, e534 10.1371/journal.pone.0000534 17579708 PMC1888724

[28] Wei J., Yu G., Shao G. et al. (2016) CYR61 (CCN1) is a metastatic biomarker of gastric cardia adenocarcinoma. Oncotarget 7, 31067–31078 10.18632/oncotarget.8845 27105510 PMC5058739

[29] Song Y.F., Xu Z.B., Zhu X.J. et al. (2017) Serum Cyr61 as a potential biomarker for diagnosis of colorectal cancer. Clin. Transl. Oncol. 19, 519–524 10.1007/s12094-016-1560-7 27743169

[30] Malhotra R.K. and Indrayan A. (2010) A simple nomogram for sample size for estimating sensitivity and specificity of medical tests. Indian J. Ophthalmol. 58, 519–522 10.4103/0301-4738.71699 20952837 PMC2993983

[31] Buderer N.M. (1996) Statistical methodology: I. Incorporating the prevalence of disease into the sample size calculation for sensitivity and specificity. Acad. Emerg. Med. 3, 895–900 10.1111/j.1553-2712.1996.tb03538.x 8870764

[32] Rice T.W., Ishwaran H., Ferguson M.K. et al. (2017) Cancer of the esophagus and esophagogastric junction: an eighth edition staging primer. J. Thorac. Oncol. 12, 36–42 10.1016/j.jtho.2016.10.016 27810391 PMC5591443

[33] Fawcett T. (2006) An introduction to ROC analysis. Pattern Recognition Lett. 27, 861–874 10.1016/j.patrec.2005.10.010

[34] Perkins G.L., Slater E.D., Sanders G.K. et al. (2003) Serum tumor markers. Am. Fam. Physician 68, 1075–1082 14524394

[35] Liu Y., Zhou Y.D., Xiao Y.L. et al. (2015) Cyr61/CCN1 overexpression induces epithelial-mesenchymal transition leading to laryngeal tumor invasion and metastasis and poor prognosis. Asian Pac. J. Cancer Prev. 16, 2659–2664 10.7314/APJCP.2015.16.7.2659 25854342

[36] Shen H., Cai M., Zhao S. et al. (2014) CYR61 overexpression associated with the development and poor prognosis of ovarian carcinoma. Med. Oncol. 31, 117 10.1007/s12032-014-0117-2 25048722

[37] D'Antonio K.B., Schultz L., Albadine R. et al. (2010) Decreased expression of Cyr61 is associated with prostate cancer recurrence after surgical treatment. Clin. Cancer Res. 16, 5908–5913 10.1158/1078-0432.CCR-10-1200 21138874 PMC3160129

[38] Wang H.Y., Hsieh C.H., Wen C.N. et al. (2016) Cancers screening in an asymptomatic population by using multiple tumour markers. PLoS ONE 11, e0158285 10.1371/journal.pone.0158285 27355357 PMC4927114

